# A promising approach to enhance microalgae productivity by exogenous supply of vitamins

**DOI:** 10.1186/s12934-017-0834-2

**Published:** 2017-11-28

**Authors:** Puja Tandon, Qiang Jin, Limin Huang

**Affiliations:** School of Environmental Science and Engineering, Shanghai Jiao Tong University, Shanghai, 200240 People’s Republic of China

**Keywords:** Renewable energy, Microalgae, Bacteria, Vitamin, Auxotroph, Symbiosis

## Abstract

In order to reduce the consumption of traditional fossil fuels and their impact on the environment, strategies to mitigate greenhouse gas emissions especially carbon dioxide needs exploration. Microalgae-based biofuels can be the best-fit plant based feed-stocks for diminishing a majority of the Universe’s energy problems. Interestingly, the eukaryotic microalgae aid in fixation of almost 50% of the global carbon in the environment. Thus, determination of parameters that will enhance microalgal growth and productivity is crucial, if they are to be used as future renewable energy sources. A large percentage of phytoplankton species are auxotroph for one or more vitamins. These species, in turn, are also dependent upon the vitamin biosynthetic pathways for processing of these vitamins. The present study serves as a base to discuss the prevalence of vitamin auxotrophy in microalgae and the methods of its acquirement from external sources such as heterotrophic bacteria. The next section of the paper sheds light on possible species-specific symbiotic interactions among microalgae and bacteria. Lastly is the discussion on how heterotrophic bacteria can act as a vitamin prototroph for an explicit microalgal vitamin auxotroph. The overall focus is placed upon harnessing these symbiotic interactions with intentions to obtain enhancements in microalgal biomass, lipid productivity, and flocculation rates. Moreover, the growth and distribution of a microalgal cell that thrives on a specific vitamin is perhaps met by growing it with the bacterial communities that nourish it. Thus, possibly by ecologically engineering a potential species-specific microalgal–bacterial consortium, it could tremendously contribute to the acceleration of photosynthetic activity, microalgal productivity, exchange of primary metabolites and other biogeochemical nutrients within the mini ecosystem. 
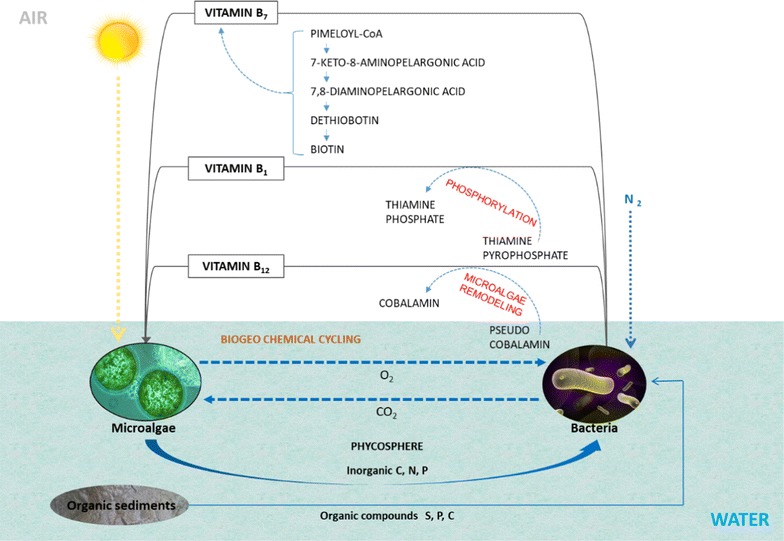

## Background

### Environmental concerns and diminishing fossil fuels

The accelerating population, impact of greenhouse gases on global warming, and depletion of fossil fuels have led to the exploration of other alternatives for renewable energy sources. The consumption of non-renewable fossil fuel energy has increased by three times over the last 50 years because of the intense development of civilization [1]. The economic engine of any civilization depends upon the production of everlasting energy resources. However, reserves of the fossil fuels are predicted to be exhausted by the first half of twenty-first century, leading to the global energy crises [2]. Nevertheless, in the consumption and depletion of these finite energy resources, we have challenged our environment and health. In order to reduce the consumption of traditional fossil fuel and as well as their impact on the environment, strategies to mitigate greenhouse gas emissions, especially carbon dioxide (CO_2_), require exploration of viable sustainable alternatives to fossil fuels [3, 4].

### Microalgae as a best-fit for future renewable energy source

Renewable and sustainable energy resources such as wind, solar, water, tidal, bioenergy (biofuels) and geothermal are being extensively generated with intentions of transiting to a clean, green, domestic and resilient renewable energy resource. Of the above resources, biofuels can be the best-fit plant based feed-stock for diminishing a majority of the Universe’s energy problems [2, 4]. Among the three generations of feed-stock (food crops, non-food crops, and microalgae) for biofuels, microalgae can be regarded as the best source of feed-stocks in terms of high photosynthetic activity, reducing food insecurity and the harmful impacts on the environment [5, 6]. The initial use of microalgae by *Homo sapiens* dates back to 2000 years when the Chinese used to cultivate *Nostoc* in order to preserve food during years of famine [7]. Lately, microalgae have received greater attention in the field of research as they have vast advantages over crop plants in terms of their ability to produce almost 300 times more renewable oil. In addition, they can better adapt to the environment, can reduce negative ecological impacts, can offer positive role in bioremediation, are commercially competitive and can be considered as an ideal organism [8]. Microalgae consist of simple unicellular and multicellular photosynthetic autotrophs. They can be either a prokaryote (cyanobacteria) or a eukaryote, with growing in different ecological environments and producing diverse metabolites [9]. Microalgae can easily grow via photoautotrophic mode by using sunlight as a solitary energy source and carbon dioxide as the carbon source through the photosynthetic reactions occurring at an optimum temperature of 25 °C [10]. Eukaryotic microalgae using photosynthetic modes can aid in fixation of almost 50% of the global carbon [11].

### Microalgal–bacterial interactions and vitamin auxotrophy

Microalgae and bacteria have existed together from the early days of evolution [12, 13]. All existing earth’s ecosystems covering the aquatic and terrestrial biomes are composed of them. Additionally, their coevolution has transformed life on earth extending from deep seas (sea sponges) to mycorrhizal fungus/lichens in all feasible modes of symbiotic associations, encompassing from mutualism to parasitism [4, 13]. Microbial associations contribute towards industrial microbiology by playing an integral part in environmental ecosystems. Some of the well-known symbiotic associations have been found between mycorrhizal fungus-plant, fungus-microalga, termite-enterobacterium, and between rhizobia–legume [14]. A greater insight into the microalgal–bacterial associations could be useful for unfolding their evolutionary and ecological roles. The knowledge of species-specific microalgal–bacterial symbiotic associations is mandatory to harness their biotechnological potential as the phycosphere microhabitat surrounding each microalga is dissimilar [12]. During the microalgal–bacterial species-specific association’s, carbon, micro-nutrients (vitamins), and macro-nutrients (nitrogen, carbon, and phytohormones) are exchanged between each other. Moreover, both the species alter their metabolism to suit each partner’s needs [15]. A complex succession of the endosymbiotic associations has led to the formation of the present dominant microalgal lineages.

Vitamins play a vital part in the development of cellular biochemistry of microalgae. However, very little information is available concerning the influence of these micronutrients on the microalgal growth, diversity, and productivity [16]. Recent research study have proven that microalgal growth can be enriched by certain growth stimulating co-factors synthesized by bacteria such as phytohormones (indole-3-acetic acid: IAA, auxin), vibrioferrin, antibiotics, vitamins, and siderophores [12]. For example, in the case of mutualistic symbiotic association between the two, bacterial species in reciprocation of fixed carbon, provide vitamin B_12_ (cobalamin) to the microalgae [17]. Some microalgae require different combinations of vitamins (biotin, cobalamin, and thiamine) as a growth factor, however; they cannot produce them. As only prokaryotes have the ability to produce some of these vitamins there has to be some definitive source of the vitamins for the microalgae.

The present study begins with a review of the prevalence of vitamin auxotrophy in microalgae and the methods of its acquirement from other micro-organisms (heterotrophic bacteria) or by exogenous supply of it from the environment. The next section of the paper sheds light on possible symbiotic interactions among microalgae and bacteria. Lastly is the discussion on how heterotrophic bacteria can act as vitamin prototroph for microalgal vitamin auxotroph is discussed. Thus, possibly by ecologically engineering a potential species-specific microalgal–bacterial consortium, it could tremendously contribute to the acceleration of microalgal productivity, photosynthetic ability, exchange of primary metabolites, and other biogeochemical nutrients within this mini environment.

### How do vitamins auxotrophy in microalgae work?

Vitamins can be defined as an organic compound and a metabolite that is crucial for the life of an organism but cannot be synthesized by them [18]. These compounds are helpful in overcoming the majority of deficiency diseases in many living entities. The organisms that cannot produce these crucial compounds but require it to be supplied to them through exogenous sources are called vitamin auxotrophs [19]. However, in many cases, these organic compounds are not required as a nutritional supplement (vitamer) as many plants, fungi, and microorganisms possess the biosynthetic pathways necessary to produce these vitamers as de nova and are called as prototrophs (synthesizers) [20]. Vitamins also act as an organic growth factor that regulates microalgal phytoplanktons growth and succession in marine ecosystems [12]. In the case of microalgae, for efficient growth, the optimized culture medium should contain nutrients, such as phosphorous, iron, sulfur, and nitrogen in addition to light, water and carbon dioxide [21]. However, for faster growth, many microalgal species also depend upon the exogenous supply of the vitamin B_12_ (cobalamin), B_7_ (biotin), or B_1_ (thiamine) in different combinations.

Table 1 [22–30] depicts vitamin requirement for different microalgal species within a phylum. More than half of the microalgal species dwelling in freshwater and marine inhabitants (156 species out of 312 species) require vitamin B_12_ in culture medium for growth, suggesting that they are auxotrophs for vitamin B_12_ [11, 16, 17], while 23% (72 species out of 312 algal species) require vitamin B_1_ and only 4.8% (15 species out of 312 algal species) require vitamin B_7_ [31]. Nevertheless, microalgal species in a phylum might have obligate requirements for one, two or all three co-factors in different combinations; though they display no correlation within any one particular lineage. However, auxotrophy is also noted amongst numerous unrelated algal phyla suggesting that it might have ascended autonomously numerous times throughout evolutions [19]. For example, within the algal group dinoflagellates, *Gymnodinium brevis* requires three vitamins while *Gymnodinium spendens* requires only one, i.e. cobalamin [19]. Similarly, genome sequences of three algal species, i.e. *Chlamydomonas reinhardtii* (Chlorophyta), *Cyanidioschyzon merolae* (Rhodophyta) and *Thalassiosira pseudonana* (Heterokontophyta) were analyzed. The first two algal species do not utilize cobalamin, while *T. pseudonana* utilizes it. In a recent research study, it was proposed that the alga, *C. reinhardtii* synthesizes cobalamin by *de nova*. In further investigations, these researchers were able to extract traces of cobalamin when the algal species were relocated from a growth medium supplemented with cobalamin to the un-supplemented growth medium. However, no genes containing sequences similar to that required for encoding cobalamin biosynthetic pathway were found after analyzing the genome of the alga. In addition, as there was no measurable vitamin in *C. reinhardtii* cell extracts, it was concluded in the specific research study that during the vitamin B_12_ biosynthesis the vitamin obtained by the algal cells was withdrawn from the supplemented growth medium [11, 17, 19].

**Table 1:** Vitamin requirement for different microalgal species within in a phylum

Algal phylum	Total species	Species requiring specific vitamin		
		B_1_	B_7_	B_12_
Chlorophyta	151	22	0	45
Rhodophyta	13	0	0	12
Cryptophyta	6	5	1	5
Dinophyta	28	7	8	24
Euglenophyta	15	11	1	13
Haptophyta	18	15	0	10
Heterokontophyta	81	12	5	47
Total	312	72	15	156
Percentage		23	4.8	50

Extracted from following references: [10, 15, 16, 18, 27, 29, 33, 63, 64]

More than half of the microalgal species expressing freshwater and marine inhabitants (156 species out of 312 algal species) have an obligate requirement for vitamin B_12_ in culture medium for growth, suggesting that they are auxotrophs for vitamin B_12_, while 23% (72 species out of 312 algal species) require vitamin B_1_ and only 4.8% (15 species out of 312 algal species) require vitamin B_7_ [31]. Nevertheless, microalgal species in a phylum might have obligate requirements for one, two or all three cofactors in different combinations; though they display no correlation within any one particular lineage

Similarly, in the case of *C. merolae*, no genes required for cobalamin synthesis were traced while in the case of *T. pseudonana* only one gene was isolated. However, the researchers were unable to detect the transcripts of that gene suggesting that it is also not expressed. They suggested that cobalamin might be synthesized by some alternative pathway similar to that present in bacteria [17, 19]. Thus, it was concluded by them that the vitamin B_12_ biosynthetic pathway was absent in these algae [19, 32].

Similarly, an interesting parallel vitamin C auxotrophy was noted in other organisms too. For example, in the case of mammals such as monkey, apes, and humans the loss of biosynthesis capability for vitamin C has occurred because of the loss of the activity of the concluding enzyme, l-gulonolactone oxidase (GULO) after 35–65 million years of evolution [33]. A similar loss in l-gulonolactone oxidase enzyme has been noted for some other lineages such as teleost fishes, passerine birds and guinea pigs indicating that auxotrophy for vitamin C occurred in these organisms because of multiple-independent damage in this gene [34].

The prevalence of vitamin (vitamin B_12_, vitamin B_1_ and vitamin B_7_) auxotrophy predominantly noticed in photosynthetic microalgae is discussed in the following sections

### Role of vitamin B_12_ (cobalamin) auxotrophy in organisms

**Fig. 1:**
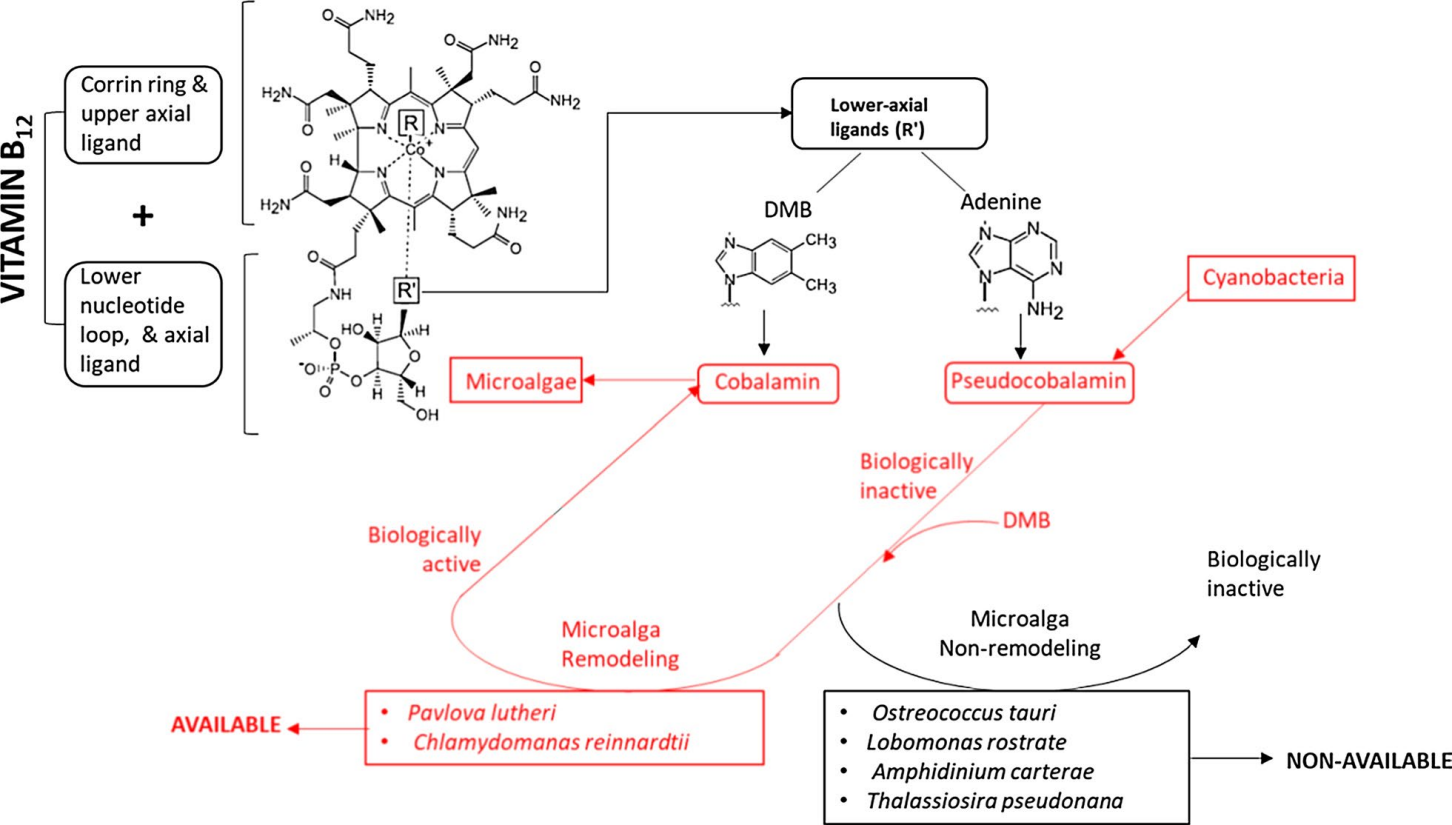
Different components in the chemical structure of vitamin B_12_ and their modes of transformation from one vitamer (chemical variant), i.e. pseudo cobalamin to another vitamer (chemical variant), i.e. cobalamin. The vitamin B_12_ consists of corrin ring plus upper ligand and lower nucleotide ring plus axial ligands attached to the cobalt ion. Certain group of microalgae such as *Pavlova lutheri* and *Chlamydomonas reinhardtii* could turn the ‘biologically inactive’ pseudocobalamin vitamer into its ‘biologically active’ form, by the term called as “microalgal remodeling”

Vitamin B_12_ also termed, as cobalamin (cobalt-containing corrinoids or ring-contracted tetrapyrroles) is a water-soluble vitamin. It is composed of corrin ring plus upper ligand and lower nucleotide ring plus axial ligands attached to the cobalt ion. Most of the organisms for growth and development require these vital molecules. Figure 1 displays different components in the chemical structure of vitamin B_12_ along with its modes of transformation from one chemical variant (pseudo cobalamin) to another (cobalamin). Vitamin B_12_ can usually be synthesized by many bacterial species, especially heterotrophic bacteria, and nearly all of the oxygenic photosynthetic cyanobacteria, but cannot be produced by eukaryotes [17, 35, 36]. Vitamins can also act as a co-factor for the enzymes that conduce methyl transfer reactions or rearrangement-reduction reactions [19]. Predominantly, vitamin B_12_ is involved in two enzymatic reactions, firstly DNA synthesis with the help of enzyme methionine synthase and secondly inorganic carbon assimilation with the help of enzyme methylmalonyl CoA mutase [36]. Several chemical variants of vitamin B_12_ are available, but they are not functionally interchangeable, e.g. between cyanobacteria and eukaryotic algae. The vitamin B_12_ chemical variant, methylcobalamin consists of the methyl group in the upper axial ligand that carries out methyl-transfer reactions. The another chemical variant, adenosylcobalamin (coenzyme B_12_) consists of adenine group in the lower axial ligand and it is involved in radical-based rearrangements and reductions [37]. However, cobalamin (chemical variant) consists of 5,6-dimethylbenzimidazole (DMB) in the base ligand. Another type of variant, pseudo cobalamin can be found in many bacterial species. Pseudo cobalamin is different from cobalamin as in its chemical structure, adenine group in the lower axial ligand replaces DMB [11]. As cyanobacteria and eukaryotic microalgae occupy freshwater and marine ecosystem, they compete with each other for light and nutrients. Pseudo cobalamin is the main type of vitamin B_12_ metabolized by the majority of cyanobacteria [38]. However, pseudo cobalamin vitamer is substantially less bioavailable compared to cobalamin to numerous vitamins B_12_-dependent microalgae, suggesting that these microorganisms cannot use it. Moreover, it is also considered as not to be “bioavailable” to humans as they have low affinity for the same in their gut [39], suggesting that they also cannot acquire the vitamer. As pseudo cobalamin consists of an alternative lower adenine base as compared with cobalamin containing the DMB ligand, it might have led to the decreased binding affinity of the adenine ligand with the microalgal METH proteins [11]. Another research study found that certain group of microalgae after a combination of pseudo cobalamin with DMB into the growth medium could convert the ‘biologically inactive’ vitamer into its ‘biologically active’ form, by the term called as “microalgal remodeling”, [11, 35]. Figure 1 also depicts the mechanism for the modification of pseudo cobalamin into cobalamin (chemical variants). The microalgal group that reacted after the addition of DMB were *Pavlova lutheri* and *C. reinhardtii* (vitamin B_12_-dependent mutant), while those that did not react were *Lobomonas rostrata*, *Amphidinium carterae*, *T. pseudonana* and *Ostreococcus tauri* [35]. Thus, it could be summarized that certain microalgal groups effectively modify the pseudo cobalamin into another vitamer such as cobalamin. This can be possible only after the reconstruction of the adenine ligand (lower axial) of the vitamin B_12_-dependent microalgae with DMB ligand, consequently modifying it to a biologically active form.

Bacteria contain more than 20 coenzyme B_12_-dependent enzymes, while the eukaryotes consist of only a few [40]. Land plants and fungi, do not possess any enzymes related to methionine biosynthesis because they contain vitamin B_12_-independent methionine synthase (METE) enzyme. However, in comparison, animals contain vitamin B_12_-dependent methionine synthase (METH) enzymes, i.e. methyl malonyl-CoA mutase (METH-CoA) and methionine synthase that is required for odd-chain fatty acid utilization and cobalamin synthesis but no METE [40]. The algal species having vitamin B_12_ auxotrophy possess no phylogenetic relationship among each other as within a species the strains might have varied B_12_ requirement [31, 41] and in certain cases, cobalamin acts as a cofactor for the enzyme methionine synthase. For example, in an early research study, it was found that *Euglena gracilis* and *T. pseudonana* [19] consists of a METH gene so in such a case cobalamin also plays a specific role in the methionine amino acid biosynthesis of the algae [42]. However, in case of *C. merolae*, it only contains METE gene, thus cobalamin is not involved in methionine amino acid biosynthesis for this algae. However, certain microalgal species, for example, *C. reinhardtii* contains isoforms for both enzymes (METH and METE), if cobalamin is available in such a case it suppresses the METE gene [43] while in its absence it suppresses the METH gene. Certain groups of researchers noted that methionine synthase is not only concerned with the biosynthesis of methionine but also in folate (vitamin) cycling for C1 metabolism [43]. Thereby, only in presence of both methionine and folate the B_12_–dependent algae can grow but not in presence of methionine alone. This phenomenon is termed as “folate-trapping” and it also occurs in humans because of vitamin B_12_ deficiency [44]. Only after the addition of folate and methionine into the growth medium at the same time, the vitamin B_12_ auxotrophy can be used by the green alga *L. rostrata*. This clearly demonstrated that trapping of folate is also prevalent in the algae and it also clarified why only partial microalgal vitamin B_12_ auxotrophy occurred even after inclusion of methionine to the growth medium [19, 45]. It also suggests that algal species can easily absorb the freely available supply of cobalamin from the environment. Furthermore, as the vitamin B_12_ auxotrophy has evolved so regularly in specific algal species, the auxotrophy could have occurred in the algae due to the loss of a specific gene that is involved in the production of the cofactor for the vitamin [17–19, 36]. Thus, the presence of a specific cofactor such as methionine synthase within the gene repertoires of the microalgae decides the actual availability of the cobalamin or it’s auxotrophy for the algal species [46]. The cell extracts of *Euglena gracilis*, *a* single celled eucaryotic algae, contains another vitamin dependent enzyme, adenosyl cobalamin dependent methylmalonyl-CoA mutase (MCM) that is required for propionate metabolism [47]. The eukaryotic alga can extract and store cobalamin, which is later transformed into two coenzymes, i.e. 5′-deoxy adenosylcobalamin (AdoCbl) and methylcobalamin. This process is diphasic, therefore involves energy independent cobalamin-binding to the mitochondrial membranes and energy dependent active transport [48]. Furthermore, in presence of light, the alga can grow by using propionate as a solitary carbon source that in turn is transformed into cell components via the MCM pathway [47, 49]. The MCM-CoA mutase enzyme in the pathway can catalyze odd-chain fatty acids and branched chain amino acids into methyl malonyl-CoA, that later is transformed into succinyl-CoA (Krebs cycle intermediate) by it. The MCM-CoA gene has also been identified in the genome of other species such as *T. pseudomona* and *Phaeodactylum tricornutum* also [19]. Remarkably, complex plastids have been found in all the species of algae that comprise of MCM-CoA. Absorption of the micronutrients such as vitamins by microalgal groups is highly challenging, as they are present in extremely low concentrations in the sea and fresh water. The amount of vitamin B_12_ in seawater ranges between 0 and 70 ng/l [50–52] followed by fresh water having little higher levels [53, 54]. However, these levels of vitamer’s are still very low in order to support vitamin B_12_-dependent microalgal growth as the microalgae require a minimum of 10 ng/l concentration of cobalamin to grow [36, 55]. This suggests that water bodies alone cannot be the only route for uptake of these micronutrients by the microorganisms. As the prokaryotes have the ability to produce the vitamins. Thus, the probable source of it to the microalgae and other organisms might be the prokaryotes themselves [19, 43]. Previous research work discussed that certain species of microalgae grow more efficiently and quickly in the presence of bacteria that produce absorbable B vitamins for the microalgae [31]. Research studies conducted in the past clearly indicate that heterotrophic bacteria [56] are effective in sustaining the vitamin B_12_ demands of microalgae and also that specific group of cyanobacteria can discharge high levels of the vitamin B_12_ in the atmosphere [57]. The outcomes from another investigation using three microalgal species proved that *C. reinhardtii*, *C. nivalis* (CCAP 11/128) and *L. rostrate* can acquire cobalamin from certain vitamin B_12_ producing bacteria [43]. For further studies on microalgal–bacterial interactions, the alga *L. rostrate* was selected amongst the two other species, since it was regarded to be a true vitamin B_12_ auxotroph. The three bacterial species, namely *Mesorhizobium loti* (strain MAFF 303099), *Rhizobium leguminosarum* (strain RL3841) and *Sinorhizobium meliloti* (strain RM 1021) representing Rhizobiales order, were heterotrophic in nature. All the three bacterial species were able to supply vitamin B_12_ to the microalgae, *L. rostrate* in the growth medium and they used the microalgal photosynthate in return, establishing a base for mutual symbiotic interactions [43]. Amongst the three microalgal–bacterial interactions, *L. rostrate*–*M. loti* association proved to achieve a steady equilibrium in terms of population sizes and time with a ratio of 1:30 microalga to bacteria. A similar sort of association was also studied between the diatom *T. pseudonana* and marine bacterium *Ruegeria pomeroyi* DSS-3 [58]. These researchers established bacterial-phytoplankton consortia consisting of these two species, wherein the diatom *T. pseudonana* acquired essential vitamin from the marine bacterium *R. pomeroyi* and the latter had an absolute dependence on the diatom for the photosynthate (carbon source). Recent research studies have proven that the growth of phytoplankton communities in the Southern Ocean becomes limited in absence of micronutrients such as iron and vitamin [16, 59]. A parallel study conducted in Chinese coastal waters reported the presence of natural and exogenous supply of vitamins in it [60] in those waters. This was further proven when a decrease in the levels of vitamin B_12_ was noticed with the increasing size of large phytoplankton, indicating the active absorption of vitamin B_12_ by this phytoplankton in South estuaries of Long island in New York [55].

It is evident that the algae do not have the capability to produce the micronutrient vitamin B_12_ de nova, suggesting that the auxotrophy due to vitamin B_12_ might have occurred owing to the absolute requirement for the vitamin cofactor essential in the microalgal metabolism and not because of its inability to produce it. However, the condition is drastically different in the case of two other vitamins, i.e. biotin and thiamine. Auxotrophy in algae due to these two vitamins was noticed because of the loss of one or more genes required for the synthesis of their co-factors.

### Role of vitamin B_1_ (thiamine) auxotrophy in organisms

The vitamin B_1_, also termed as thiamine (thiamine pyrophosphate), is a colorless organosulphur. It is a water-soluble compound, biotically synthesized and nitrogen containing catalyst having a chemical formula of C_12_H_17_N_4_OS. The vitamin B_1_ contains an aminopyrimidine and a thiazole ring connected via a methylene bridge. The vitamin B_1_ co-factor was the first cofactor that was noted to be involved in influencing microalgal growth [19]. Moreover, the word ‘vitamin’ or ‘vital amine’ was designated only after the discovery of vitamin B_1_ [18]. After phosphorylation of thiamine phosphate, an active co-factor thiamine pyrophosphate (TPP) is produced. The cofactor can be found in processed and whole foods and plays a major role in carbohydrate metabolism (glycolysis). Only those microorganisms are able to synthesise thiamine that contain complete thiamine metabolic pathway, while the others depend upon external sources such as symbiosis or scavenging, to meet their vitamin B_1_ quota [61]. Thiamine in the form of TPP acts as a co-factor for a large number of enzymatic reactions, for example during carbohydrate metabolism and amino acid synthesis it is required in pyruvate dehydrogenase complex [36]. Similar to vitamin B_12_, it is required by a large number (22%) of phytoplankton for growth. For example, almost 73% of euglenophytes and 83% of prymnesiophytes from algal species require an external supply of thiamine [16]. In earlier studies, the amount of vitamin B_1_ detected in the natural marine ecosystem at different locations in the Pacific Ocean typically ranged between 8 and 15 ng/l [50, 51, 62] which is far below the level of vitamin required by the algal species. Moreover, at an alkaline pH of seawater, and temperature ranging between 10 and 30 °C, a steady decline in the stability of thiamine was noted, suggesting that the co-factor for this vitamin is not acquired by marine microalga from natural sea waters.

Previous studies have confirmed that this vitamin plays a critical role in enhancing the primary productivity of marine ecosystems, microalgal progressions and induction of microalgal blooms [63, 64]. Other studies have found a positive correlation amongst primary productivity and increase in vitamin B_1_ concentration in subarctic North Pacific seawater [50, 51]. But, the bioavailability of this vitamin to the microalgae is in turn dependent upon multiple aspects of microalgal cell physiology, such as utilization forms, uptake affinity and percentage requirements per cell [65]. Earlier studies carried out on the specificity of the thiamine requirement indicated that in certain marine microalgal species the auxotrophy could be fulfilled by the thiazole or pyrimidine moieties and/or by its chemical analog’s (chemical compounds having structural similarity to vitamin B_1_) addition to the culture medium [65]. For instance, the microalgal strain of *Emiliania huxleyi* is able to utilize both vitamin B_1_ and/or 4-amino-5-hydroxymethyl-2-methylpyrimidine (HMP) in order to satisfy its thiamine requirement. However, in other cases, two strains of *Micromonas* had an obligate requirement for vitamin B_1_ and were not able to utilize its chemical analogs in order to fulfill its thiamine requirement [66]. The thiamine biosynthetic pathway in most of the microalgal vitamin B_1_ auxotrophs exhibits similar patterns as found in other organisms. Herein, two separate branches, one of thiazole ring and another of pyrimidine ring comprise of independent branches that after combination give rise to thiamine. Additionally, the occurrence of sub parts of some of these thiamine biosynthetic pathway in microalgal thiamine auxotrophs depicts that thiamine is essential for these organisms, however, during the course of evolution some of the necessary genes involved in its biosynthesis have been lost [19]. Remarkably, no phylogenetic resemblances have been found amongst different vitamin B_1_ auxotrophs (non-synthesizers), demonstrating that the loss of the genes capable of biosynthesis might have evolved multiple times [18]. Moreover, another set of researchers found that in the case of a model green alga, *C. reinhardtii* which can be regarded as a vitamin B_1_ prototroph (synthesizer), the physiological concentrations of thiamine in the biosynthetic pathway can be self-regulated within the cell by the use of riboswitches [20]. The riboswitches are small 5′ end bio sequences in the mRNAs wherein the co-factors such as thiamine pyrophosphate (TPP) binds, thus altering the translation initiation and/or premature transcription termination and thereby regulating the enzymes involved in the thiazole and/or pyrimidine moieties [20, 67]. Additionally, in the case of other algal species such as *T. pseudonana*, and *C. merolae*, thiamine or any of its biosynthetic pathway intermediates are not required for their growth, depicting *de nova* synthesis of these vitamins by these species [19]. The similar pattern was also observed in some picocyanobacterial (*Synechococcus* and *Prochlorococcus*) phytoplankton species that contained the complete genome for vitamin B_1_ biosynthetic pathway and, was in turn, not dependent upon any exogenous/external environmental vitamin B_1_ source [38, 65]. However, in severe contrast, many picoeukaryotic phytoplankton species are dependent upon exogenous/external environmental vitamin B_1_ source [65].

In vitro studies have confirmed that thiamine displays antioxidant activities [68] and is possibly also connected to the accumulation of the reactive oxygen species, i.e. hydrogen peroxide (H_2_O_2_) in the plants [69]. As compared to other phytoplanktons, thiamine has also been noted to be a significant part of a cellular oxidative stress defense system in chlorophytes such as *Ostreococcus* and *Micromonas* spp. This was confirmed by the fact that the demand for vitamin B_1_ as a cofactor for enzymes in these green algae, described as minimum vitamin B_1_ cell quotas, were more than as required by other phytoplanktons [65]. Other studies noticed almost 2–6-fold enhancements in microalgal growth, elevation in nutrient removal rates and doubling of lipid content (neutral) after co-culturing a green alga *Auxenochlorella protothecoides* with a bacterium *Escherichia coli* demonstrating a symbiotic relationship. Further experiments conducted by the same group proved the concept of symbiosis among the two as the bacterium transferred the thiamine derivatives (thiamine pyrophosphate, thiamine precursor, and HMP) and degradation products (extracted from the residual cell-free medium) necessary for algal growth in return of algal photosynthate [70].

### Role of vitamin B_7_ (biotin) auxotrophy in organisms

Vitamin B_7_ also termed as biotin and formerly known as Vitamin H is a colorless, water-soluble member of the B vitamin group. It plays a critical role as a co-factor in carbon dioxide metabolism for various carboxylases enzymes [71]. These enzymes, in turn, are involved in numerous metabolic processes such as gluconeogenesis, citric acid cycle, regulation of gene expression, branched chain amino acid catabolism and fatty acid biosynthesis [72]. Biotin heterocyclic system consists of an ureido branch merged with a tetrahydrothiophene (thiophane) branch. Additionally, a substituent of valeric acid is fused with one of the thiophane branch [71, 73]. In earlier studies, the concentration of biotin detected in natural waters varied between 1 and 4 ng/l at various locations in the Pacific Ocean [50, 51, 62]. However, it is quite below the concentration of vitamin required by the algal species. In contrary, another research study have noted that the concentration of biotin in surface water varies between 0.1 and 57.9 ng/l, wherein mostly lower concentrations in deep oceans and high concentrations in coastal waters were noted [74, 75]. Furthermore, they noted concentrations were high during summer months and decreased rapidly during winter and autumn months.

Certain groups of microalgae require biotin in small amounts either in bound form or free state from exogenous sources. Data extracted by researchers found that from 306 algal species surveyed till date, only 14 were auxotrophs for biotin [19]. Altogether, these auxotrophs, such as *Ochromonas danica* (heterokontophyte), *Eutreptia* sp. (Euglenophyta), *Amphidinium klebsii* (dinophyte) and *Gyrodinium cohnii* (dinophyte) belong to the algal groups with complex plasmids that require the vitamin for growth stimulation [19, 30]. However, every biotin auxotroph has a demand for two other vitamins, i.e. either both thiamine and cobalamin or only one of them, however, in different combinations [76, 77]. Other algal species such as *C. merolae*, *C. reinhardtii*, and *T. pseudonana* are biotin prototrophs, as they possess numerous biotin-dependent carboxylases, that might be involved in the processing of the vitamin through the functional metabolic pathway [17]. The examples of multiple independent gene loss have also been noted across various lineages in the case of biotin. A previous study isolated 44 biotin synthesis related genes from 14 photosynthetic algal genomes belonging to three groups, i.e. Chlorophyta-green algae (10), Rhodophyta-red algae (1), Prymnesiophyta (haplophyta) (1) and/heterokonphyta-diatoms (2) [72]. These 44 biotin biosynthesis related genes can synthesis biotin from pimeloyl-CoA through four step processes that are catalyzed by enzymes such as 7-keto-8-amino pelargonic acid synthase (KAPA), 7,8-diamino pelargonic acid synthase (DAPA)/dethiobiotin synthase, and biotin synthase, however, its source differs amongst different species [78]. Additionally, another group of researchers discussed that the enzymes involved in the conversion of DAPA to dethiobiotin synthase in alga are yet to be identified [19]. It was also specified by them that perhaps the source of dethiobiotin in the alga might have been via bacteria through their unanticipated symbiotic associations [72]. Previously, similar types of associations for biotin transfer have also been noted amongst alga and fungus in the lichen *Peltigera aphthosa* [33, 79–81]. However, many lineages cannot synthesize biotin even though they possess certain genes required for its synthesis [18]. Furthermore, different biosynthetic pathways have been followed by different lineages in order to synthesize the vitamins. Even though different lineages possess alternative biosynthetic routes, still similarities are found in sources and processes leading to this loss. Altogether, it could be said that biotin auxotrophy might have been caused by the loss of some genes required for the biosynthetic pathway; this gene could be different amongst various lineages, however.

The three vitamins discussed above are vital for the growth of many photosynthetic microalgae. Despite this, the role of the vitamin, its source and synthesis remain a topic of debate. The knowledge of the vitamin biosynthetic pathway is essential for processing of these vitamins. Additionally, more light needs to be shed on the ecological role of these vitamins in the marine ecosystem.

### Symbiotic interactions: bacteria as exogenous sources of vitamins

The co-evolution of vitamin-biosynthetic pathways via inclusion or loss of specific gene instances have directed in a specific variation of auxotrophy amongst different lineages [18]. Moreover, the intricate pattern of vitamin auxotrophy across and within different lineages has led to display the interdependence of these lineages amongst each other. Many microorganisms (vitamin auxotrophs) require an external source of the vitamins, as they cannot synthesize it by themselves. These microorganisms, in order to obtain the vitamins from their immediate surroundings, will exhibit intimate complex interactions with other organisms. A greater insight into these types of interactions amongst organisms employing exchange of nutrients or growth promoting factors by both collaborators (symbiosis) or by one partner (commensalism/parasitism) in natural biomes, is a vital aspect that has been addressed less rigorously. Moreover, these interactions collectively impart a positive effect on the global carbon cycle and biogeochemical cycling in the environment, thus reducing global warming.

### Symbiotic interactions: mutualism and commensalism

There is strong evidence of a number of biological interactions amongst two or more partners of different lineages aiding each other [12, 13]. Microalgal phytoplanktons in some environmental ecosystems dwell in an intimate relationship with symbiotic bacteria that pioneer the phycosphere and prosper on the metabolites released by the microalgae and undergo a diversity of activities [82]. The collective interaction between both partners is a case of mutualistic symbiotic interaction. For example, the symbiotic interactions between rhizobia–legume, termite–enterobacterium, fungus–microalga and microalgae–bacteria [5, 14]. Table 2 [22, 27, 28, 34, 80, 83–88] illustrates various examples of symbiotic interactions between different microorganisms for the exogenous supply of vitamins. The interactions between the microorganisms range from specific mutualism to commensalism depending upon the species and the environmental aspects that influence the mini ecosystem. For instance, in the case of mutualistic symbiosis between a bacterial species, *Halomonas* sp. and marine red microalgal species, *Porphyridium purpureum*, wherein the bacterial species provides cobalamin to the microalgal associate in exchange for algal photosynthate [17]. In a distinct microalga–bacterial consortium the potential sources of symbiosis could be:

**Table 2:** Examples of symbiotic interactions between different microorganisms for exogenous supply of vitamins

Association	Algae	Bacterium/fungus	Intermediaries from algae	Intermediaries from bacterium/fungus	Vitamin concentration	Reactions carried by the vitamin	References
Mutualism and commensalism	*Porphyridium purpureum*	*Halomonas* sp.	Carbon source and algal extracts	Vitamin B_12_	10 ng/l	–	[16]
	*Thalassiosira pseudonana*	*Ruegeria pomeroyi* DSS-3	Dihydroxypropanesulfonate-3-dehydrogenase (DHPS; 3.3 mM) as carbon source	Vitamin B_12_	–	DNA Synthesis by enzyme methionine synthase	[56]
	*Lobomonas rostrata*	*Mesorhizobium loti* MAF	Algal photosynthate	Vitamin B_12_	60 ng/l	Inorganic carbon assimilation by enzyme methylmalonyl COA mutase	[80]
	*Chlamydomonas reinhardtii* CC23	*Mesorhizobium loti* (heterotrophic bacteria)	No exchange of photosynthate	Vitamin B_12_	100 ng/l	–	[41]
	*Eutreptiella* sp.	Ectobiotic bacteria (*Marinobacter*) and endobiotic bacteria	Bacteria uses host cells to reproduce	Vitamin B_12_ and growth promoting factors	1 mg/l	–	[82]
	*Auxenochlorella protothecoides*	*Escherichia coli*	Algal photosynthate	Vitamin B_1_, 4-amino-5-hydroxymethyl-2-methylpyrimidine (HMP) and degradation products	20 ng/l	Pyruvate dehydrogenase complex used for carbohydrate metabolism and amino acid synthesis	[27]
	*Ostreococcus lucimarinus* CCE9901	*Pseudoalteromonas* sp. TW7	Algal photosynthate	Vitamin B_1_ and modify vitamin analogs	77.7 ng/l	–	[63]
	*Coccomyxa* sp.	*Peltigera aphthosa*	Host tissue	Biotin	7.2 m µg per milligram	Cofactor in carbon dioxide metabolism in various carboxylases enzymes	[76–78]
Parasitism	*Chlorella vulgaris*	*Exophiala* sp. (*fungus*)	Host	Perform algicidal activity	–	–	[89]
	*Emiliania huxleyi*	*Phaeobacter gallaeciensis*	Mutual/host	Roseobacticides A and B		Roseobacticides A and B, tropodithietic acid (TDA), thiotropocin (safeguarded the algal cells) and phenylacetic acid (enhanced algal growth)	[85]
	*Prorocentrum minimum*	*Dinoroseobacter shibae DFL*-*12*	Mutual/host	Vitamin B_12_ and roseobacticides	–	–	[79]

The interactions between the microorganisms range from specific mutualism to commensalism to parasitism depending upon the species and environmental factors of the mini ecosystem. A typical example of mutualistic symbiosis occurred between a bacterial species, *Halomonas* sp. and marine red microalgal species, *Porphyridium purpureum* wherein the bacterial species supplies cobalamin to the microalgal associate in exchange for fixed carbon

- Primary metabolites (carbon dioxide-oxygen exchange) [89],
- Growth co-factors (vitamin) and phytohormones; auxins, cytokinins [19, 90], and,
- Recycling of nutrients such as phosphorous and nitrogen after formation of distinct physical niches/biomes in an aquatic ecosystem [70, 91].

In another ecosystem, ectobiotic bacteria (*Marinobacter*) and endobiotic bacteria were associated with microalga *Eutreptiella* sp., wherein the ectobiotic bacteria provided cobalamin and growth promoting factors to the alga and in turn, the endobiotic bacterial cells were able to reproduce within the host cells [86]. A recent research study inspected the growth dynamics among the populations of the interacting organisms in an aquatic ecosystem in order to understand the mechanisms involved in these interactions and in building a similar ecosystem for industrial purposes. They illustrated a simple two-organism ecosystem in which the bacterium *Mesorhizobium loti* provides cobalamin to the green freshwater microalga *L. rostrata* in exchange of algal photosynthate [84]. Other studies demonstrated laboratory culturing of a simple consortium consisting of a bacterium, *Pseudoalteromonas* sp. TW7 (thiamine prototrophic bacteria) and marine picoeukaryotic algal strain *Ostreococcus lucimarinus* CCE9901 (thiamine auxotroph). In this case, it was observed that bacteria can improve the vitamin bioavailability and consumption of thiamine auxotrophic alga thru *de nova* synthesis of the thiamine analogs [65]. Recently, bacteria were termed as “producers” of vitamins. They also possess the capability to chemically modify the vitamin to make it bioavailable to the microbes that cannot use the vitamin analogs by themselves [65]. In another set of experiments almost 2–6-fold stimulation in growth was noted for green alga, *Auxenochlorella protothecoides* co-cultured with a bacterium, *Escherichia coli* under mixotrophic conditions. Herein the concept of symbiosis was again proven between the two species as the bacterium provided thiamine derivatives and degradation products to the green algae in exchange of oxygen and primary metabolites [70]. In another study, in the case of biotin auxotrophy, it was discussed that maybe the external source of dethiobiotin in the microalgal species might be bacteria via unanticipated symbiotic interactions among both of the species [19]. Another study promoted the formation of such beneficial symbiotic interactions among the algae and fungi, as in the case of lichen, *Peltigera aphthosa* for the exchange of dethiobiotin synthetase/adenosyl methionine-8-amino-7-oxononanoate aminotransferase amongst them [72, 80]. However, commensalism is a type of biological interaction in which only one-partner benefits from the association. For example, the relationship in between green microalga *C. reinhardatii* and heterotrophic bacteria, wherein the microalga utilizes cobalamin provided by the bacteria, however, the bacteria does not utilize microalgal photosynthate (organic carbon source) from the microalgae [43]. According to the information gathered in Table 2, it could be summarized that species-specific mini ecosystem could be constructed to obtain desirable results. Additionally, there is a narrow difference between the concepts of mutualism and commensalism, or parasitism as the change in environmental parameters might shift one category to another.

### Symbiotic interactions: parasitism

Parasitism is a relatively well-studied concept in which one partner prospers on the compounds secreted by the other partner or competes with the later for the micronutrients, thereby exerting negative pressure on the later [5, 13, 82]. In most of these interactions, the parasite is smaller and requires a living host, except in the case of a certain group of algae (red) that dwell upon partners of higher taxa or their counterparts [12]. For example, after culturing an ecologically engineered microalgae-bacterial/fungal phycosphere in a laboratory using *Microbacterium* sp. (bacteria)/*Exophiala* sp. (fungus) and a green microalga *Chlorella vulgaris*, reduction in microalgal growth was noted. The *Microbacterium* sp. has been previously reported to compete with microalga for nutrients and slow down its growth, however *Exophiala* sp. releases certain anti-bacterial compounds or perform algicidal activity thereby inhibiting microalgal growth [92]. In another study, mutualistic interactions amongst marine microalga, *E. huxleyi* (environmentally important alga) and a roseobacter clade, i.e. *Phaeobacter gallaeciensis* were studied [88]. The researchers found two diverse phases in the life cycle of roseobacter–microalgal association. In an initial phase, when the microalgal host cells are young, it forms a mutual symbiotic relationship with the bacteria. The bacteria provided broad-spectrum antibiotics tropodithietic acid (TDA) in addition to thiotropocin that safeguarded the algal cells from bacterial pathogenesis and phenylacetic acid that enhanced algal growth [93]. In a latent phase in response to higher concentrations of p-coumaric acid produced during microalgal senescence, *P. gallaeciensis* released roseobacticides A and B (algicides called roseobacticides) from TDA and thiotropocin that caused algal cell wall lysis. This switch changed *P. gallaeciens* from a mutualistic partner to a pathogenic partner, proposing a “Jekyll and Hyde” type of hypothetical lifestyle of *Roseobacter*s [82, 88]. In another study, the photoheterotrophic *Dinoroseobacter shibae* DFL-12 (*Roseobacter clade*) and toxic phototrophic dinoflagellate *Prorocentrum minimum* were cultured together. In the mutualistic phase, the bacteria acted as vitamin B_12_ prototroph and synthesized it de nova as well as provided it to the dinoflagellate. In the latent phase, the same bacteria inhibited and starved the dinoflagellate cells to death [34, 94] following the same “Jekyll and Hyde” type of hypothetical lifestyle of *Roseobacters* [82]. The roseobacter associated lineages have the potential to be exploited extensively in order to serve as an excellent mutualistic partner for microalgae in an aquatic ecosystem as they not only provide the latter with vitamins and phytohormones but also safeguard them via releasing antibacterial compounds from other non-roseobacter species [95].

The selective burden on the biosynthetic pathway of the vitamin auxotroph can be reduced by the availability of the vitamer in required quantities from external resources. The specific auxotroph has evolved its associations with other lineages in order to deal with these pressures and fluctuations leading to vitamin out-sourcing and trafficking amongst their communities. Microalgal–bacterial synergetic associations might be responsible for governing the exchange of primary metabolites, growth co-factors and major compounds within the ecosystem. More precisely, these species-specific beneficial associations could underpin most of the microalgal features in order to obtain better productivity.

### Conclusion and future aspirations

In order to compete with existing fossil fuel resources, the economic parameters of producing microalgal biofuel have to be enriched extensively with intentions to decrease its harvesting cost and enrich its productivity. An adequate supply of dissolved vitamins is vital for the growth of phytoplanktons, especially microalgae in an aquatic ecosystem. However, the inclusion of these vitamins in the growth medium or its absorbance from the aquatic environment has led to an acceleration of photosynthetic activity of a large number of phytoplankton communities. A large percentage of phytoplankton microalgal species are auxotrophs for one or more vitamins. In addition, these species are dependent upon the vitamin biosynthetic pathways for acquiring and processing of these vitamins. Microalgal vitamin auxotrophy, in the case of cobalamin, might have occurred due to the obligate requisition of the same during microalgal metabolism and not because of its incapability to produce the micro-nutrient. However, this is altogether different for auxotrophy for thiamine and biotin that seems to have occurred due to the deficiency of some genes involved in the processing of their co-factors. In an ecological ecosystem, the growth and distribution of a microalgal cell that thrives on a specific vitamin is largely dependent upon the bacterial communities that nourish it. Ecologically engineering a potential species-specific microalgal–bacterial ecosystem could tremendously contribute to the acceleration of photosynthetic activity, microalgal lipid productivity and growth, exchange of primary metabolites and other biogeochemical nutrients within this mini ecosystem. The improved microalgal fuel can collectively influence the global carbon cycle by decreasing the concentration of greenhouse gases within the environment, lowering the microalgal production cost by minimizing the input of micronutrients in the culture medium, and aiding in faster biomass recovery.

## Abbreviations

AdoCbl: 5-deoxyadenosylcobalamin
DAPA: 7,8-diamino pelargonic acid synthase
DHPS: dihydroxypropanesulfonate-3-dehydrogenase
DMB: 5,6-dimethylbenzimid azole
GULO: l-gulonolactone oxidase
HMP: 4-amino-5-hydroxymethyl-2-methyl pyrimidine
IAA: indole-3-acetic acid
KAPA: 7-keto-8-amino pelargonic acid synthase
MET-CoA: methylmalonyl-CoA mutase
METE: vitamin B_12_-independent methionine synthase
METH: vitamin B_12_-dependent methionine synthase
TDA: tropodithietic acid
TPP: thiamine pyrophosphate
